# Coregulation mapping based on individual phenotypic variation in response to virus infection

**DOI:** 10.1186/1745-7580-6-2

**Published:** 2010-03-18

**Authors:** German Nudelman, Yongchao Ge, Jianzhong Hu, Madhu Kumar, Jeremy Seto, Jamie L Duke, Steven H Kleinstein, Fernand Hayot, Stuart C Sealfon, James G Wetmur

**Affiliations:** 1 Center for Translational Systems Biology and Department of Neurology, Mount Sinai School of Medicine, New York, NY 10029, USA; 2 Department of Microbiology, Mount Sinai School of Medicine, New York, NY 10029, USA; 3 Interdepartmental Program in Computational Biology and Bioinformatics and Department of Pathology, Yale University, New Haven, Connecticut 06511, USA

## Abstract

**Background:**

Gene coregulation across a population is an important aspect of the considerable variability of the human immune response to virus infection. Methodology to investigate it must rely on a number of ingredients ranging from gene clustering to transcription factor enrichment analysis.

**Results:**

We have developed a methodology to investigate the gene to gene correlations for the expression of 34 genes linked to the immune response of Newcastle Disease Virus (NDV) infected conventional dendritic cells (DCs) from 145 human donors. The levels of gene expression showed a large variation across individuals. We generated a map of gene co-expression using pairwise correlation and multidimensional scaling (MDS). The analysis of these data showed that among the 13 genes left after filtering for statistically significant variations, two clusters are formed. We investigated to what extent the observed correlation patterns can be explained by the sharing of transcription factors (TFs) controlling these genes. Our analysis showed that there was a significant positive correlation between MDS distances and TF sharing across all pairs of genes. We applied enrichment analysis to the TFs having binding sites in the promoter regions of those genes. This analysis, after Gene Ontology filtering, indicated the existence of two clusters of genes (CCL5, IFNA1, IFNA2, IFNB1) and (IKBKE, IL6, IRF7, MX1) that were transcriptionally co-regulated. In order to facilitate the use of our methodology by other researchers, we have also developed an interactive coregulation explorer web-based tool called CorEx. It permits the study of MDS and hierarchical clustering of data combined with TF enrichment analysis. We also offer web services that provide programmatic access to MDS, hierarchical clustering and TF enrichment analysis.

**Conclusions:**

MDS mapping based on correlation in conjunction with TF enrichment analysis represents a useful computational method to generate predictions underlying gene coregulation across a population.

## Background

Variability in human immune response is a well known fact, confirmed each year when the seasonal influenza arrives. Understanding why individuals show a diversity of immune responses would have important clinical implications. Investigation of the role of functional single nucleotide polymorphisms (SNPs) in immune related genes in the response to challenge by infectious agents is part of the effort to study variability in the human immune response [1,2]. Here we investigate human variability at a cruder level, the level of gene expression across a group of individuals infected by the same virus. We are looking at correlations and their basis among a number of genes upregulated as a result of the infection. The infection is by Newcastle disease virus (NDV), an avian parainfluenza virus that lacks the tools to disrupt the human innate immune response, and therefore engages it fully. The cells are human dendritic cells (DCs), which are important players in innate immunity and initiators of adaptive immunity through T cell activation. The study of gene correlations in a population, though mathematically similar, is very different from the study of a data set where gene expression levels would be measured at a number of time points after infection for cells from the same individual. Here the variability is found across humans themselves not in the time courses of expression levels [3,4].

Our data show considerable variability in human immune response for DCs infected by NDV. In order to uncover the underlying patterns of gene expression, we studied gene correlations across the population, with the aim of clustering genes according to some distance measure. There are many distance measures reported in the literature and available via web based computational analysis tools. Our goal was to obtain a combination of distance measure and transcription factor (TF) binding site analysis that provided an efficient methodology for both clustering genes and investigating their coregulation. We used a combination of established methods ranging from multi-dimensional scaling (MDS) to the evaluation of Jaccard coefficients for TF sharing, to a formula for a global TF enrichment score, that cannot be found jointly at present on the well-known web server Gene Pattern [5]. For our type of investigation, namely the correlation of a small number of gene expression levels across a human population, MDS is a very useful method. It is more flexible than principal component analysis (PCA) [6,7], since it can be implemented for any type of correlation coefficient calculation, in particular for Kendall's which, because it is based on relative rank, provides the least constrained way of calculating correlations. Though hierarchical clustering [8-10], which we also give for completeness, can be derived for any correlation coefficient, it does not exhibit, because of its tree like structure, the rich two dimensional organization of a MDS distance scheme. Though MDS has been applied to large data sets [11], we use it here for a small set of 34 genes, which are further reduced in number through considerations of experimental procedures and GO category filtering. Our methodology is helpful in exploring hypotheses about gene coregulation.

## Results and Discussion

### Response Variation Dataset

**Figure 1:**
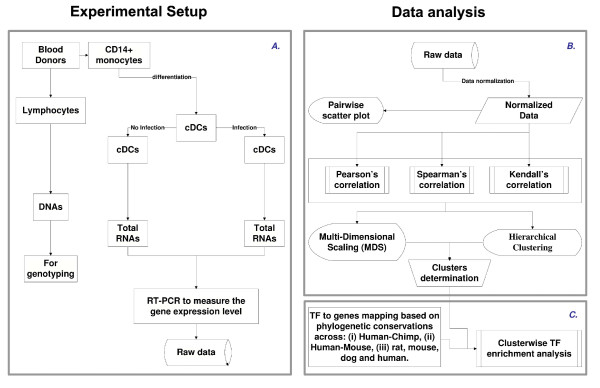
**Flowchart illustrating the overall methodology**. **A**. Experimental design. **B**. Illustration of an approach to systematically identify and represent the correlation among the different genes across the population. **C**. Illustration of TF prediction procedure. Note that the procedure was developed in a modular form, which allows robustness and therefore can be used in the web tool.

**Figure 2:**
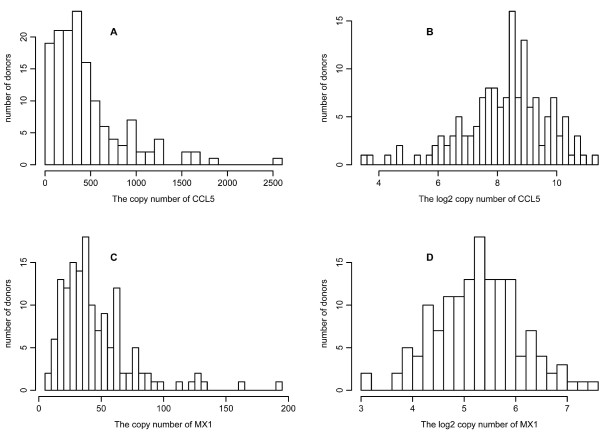
**Variation in gene response**. All qPCR measurements for 34 genes were taken after the DCs were infected with NDV for 10 hours. Results shown are for CCL5 (**A **and **B**) and MX1 (**C **and **D**). The histograms are drawn for the copy number (**A **and **C**) and log_2 _copy number (**B **and **D**) of the corresponding mRNA from 145 donors.

We began with the set of genes previously identified to participate in the pathways involved in the innate immune response to NDV infection of human DCs [12,13]. We then used our microarray data from NDV infected primary human DCs [14] to identify a panel of 34 significantly induced key regulator genes from this set. The microarray study suggested that the expression of most early induced genes reached a plateau by 10 h after infection. The experimental methodology is summarized in Figure 1A. In Figure 2 we show typical histograms of two of the measured genes, a chemokine CCL5 and an antiviral protein MX1, that illustrate the fact that gene expression levels can vary considerably among individuals, varying on the log2 plots by several units for CCL5 and MX1. Moreover the levels at which genes are expressed vary as well significantly, with copy numbers up to 100 for MX1, but up to 1000 for CCL5 (Figure 2). The raw data and a summary table are presented in Tables S1 [see Additional file 1] and S2 [see Additional file 2], respectively. As the copy number data were not well fitted by Gaussian or other parametric distributions (Figures 2A and 2C), we computed the median and the median absolute deviation (MAD), which gave robust estimates of the center and the spread of the distributions. For a Gaussian distribution, the median is the same as the mean, and the MAD the same as the standard deviation. We transformed the copy number by the log_2 _function so that the distribution could be approximated by a Gaussian (Figures 2B and 2D). The data for log_2 _copy numbers are summarized by the mean and standard deviation in Table S2. The variation contributed by the experimental procedure (Table S2) is estimated by repeating the same experiment six times on the same individual donor.

We filtered out genes showing too low gene expression (gene names in blue with a crossed line in Table S2, copy number<2), and kept the genes with experimental variation  smaller than the donor population variation  (colored in red in the last column of Table S2) at some statistical level. The variance comparison was done by an F-test with H_0_:  vs H_1_: . Of the original list of genes, we retained the 13 with copy number greater than 2 and with an uncorrected p-value smaller than 0.05 for the F-tests. The highest false discovery rate [15] for the 13 genes is 10.5%. Because the effective multiplicity of infection (MOI) defined as the virus/cell ratio may vary among the experiments, we also measured the expression of viral RNA. We examined the effect of adjustment of the gene copy numbers by dividing by NDV expression, which gave a larger list of 27 genes with p-value no greater than 0.05 for the F-test. However, some genes are induced only in infected cells whereas others are expressed similarly in both infected and uninfected cells. The aforementioned NDV correction procedure may introduce bias for the genes whose expression is not directly related to the MOI. The 13 genes that are selected from the data without NDV correction also appear significant in the data after NDV correction. To be conservative, we used the unadjusted 13 filtered genes for our subsequent analysis.

### Correlation Mapping

We hypothesized that genes showing similar patterns of change across infected individuals would be likely to share common genetic mechanisms responsible for these patterns. Our first goal was therefore to develop an approach to systematically identify and represent the correlation among the different genes across the population. We tested several metrics for pairwise correlation and represented the results using multidimensional scaling and, for completeness, hierarchical clustering. The approach is shown schematically in Figure 1B.

**Figure 3:**
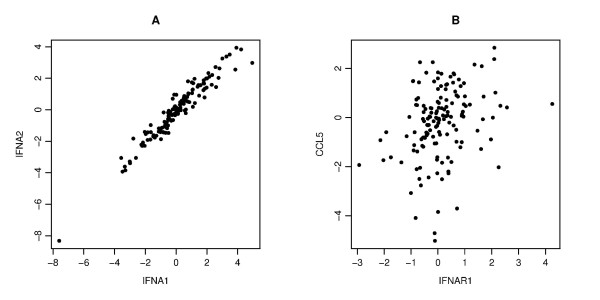
**An example of the scatter plots for two pairs**. Each panel gives a scatter plot of a pair of genes. The x-axis and y-axis give the log_2 _transformed values of the corresponding gene copy number. Panel **A **displays a strong correlation between IFNA1 and IFNA2 (Pearson's, Spearman's and Kendall's correlation coefficients are r = 0.964, ρ = 0.959, τ = 0.837, respectively), Panel **B **displays a weak correlation between IFNAR1 and CCL5 (r = 0.288, ρ = 0.317, τ = 0.223).

We started by examining scatter plots comparing the expression levels of pairs of the 13 genes under consideration from the 145 donors. These showed varying levels of correlation (Figure 3). For example, the levels of IFNA1 and IFNA2 were strongly correlated (Figure 3A), whereas IFNAR1 and CCL5 were weakly correlated (Figure 3B). For quantification, we evaluated three types of correlation coefficients, Pearson's, Spearman's and Kendall's (Table S3; see Additional file 2). Pearson's correlation reflects a linear relationship between the levels of the two genes. Spearman's correlation is computed by replacing the data points with their rank and captures monotonic correlations. Kendall's correlation is less constrained than Spearman's as it uses relative rankings only between pairs of data, which makes it the most robust correlation measure of the three (See reference [16] and the Methods section for details of the calculation of these correlations). The results of this analysis showed that the pairs of genes ranged from strongly correlated to weakly correlated (Table S3). It is worth noting that correlation coefficients results were similar for all three.

**Figure 4:**
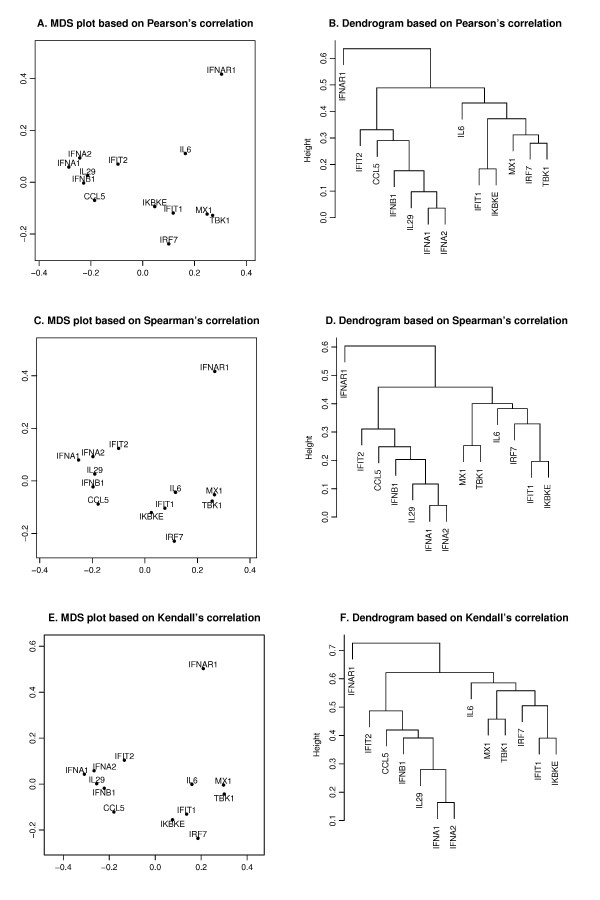
**Pearson's, Spearman's and Kendall's correlations in MDS and hierarchical clustering**. The MDS plots (**A, C, E**) and dendrograms of hierarchical clustering (**B, D, F**) of the 13 genes based on a different correlation metric. For the MDS plot, the x-axis and y-axis give the coordinates of the 13 genes chosen to represent the dissimilarities of the genes among 145 samples. For the hierarchical clustering, the y-axis (height) indicates the dissimilarity between two clusters of genes. The dissimilarity is based on one minus the correlation coefficients of normalized log_2 _copy numbers, where the correlation coefficients can be Pearson's (**A, B**), Spearman's (**C, D**) and Kendall's (**E, F**).

We next used the correlation matrices (Table S3) to visualize the cluster structure of the genes either through hierarchical clustering dendrograms or multidimensional scaling (MDS) (Figure 4) For the latter we generated two-dimensional maps using the pairwise dissimilarities (one minus the correlation) as a distance measurement.

We briefly describe the use of MDS for gene correlation clustering visualization. Let δ_*ij *_be the dissimilarity between gene i and gene j. The MDS finds the representative coordinates (x_i_, y_i_) of the 13 genes such that the stress function,

is minimized, where *d*_*i*, *j *_is the geometrical Euclidean distance between the representative points (x_i_, y_i_) and (x_j_, y_j_). Further details of the MDS algorithm can be found in reference [17]. The geometric distance between any pair of genes reflects the dissimilarity in relative levels between the two genes among the 145 samples (Figure 4). The MDS approximation is relatively good, as given by the stress function 0.075, 0.092 and 0.17 for the Pearson's, Spearman's and Kendall's coefficients, respectively. The analysis for the three measures of dissimilarity showed the grouping of two clusters: (CCL5, IFNA1, IFNA2, IFNB1, IFIT2, IL29) and (IFIT1, IKBKE, IL6, IRF7, MX1, TBK1).

The MDS analysis can also be applied to the 145 samples and the 6 experimental repeats of a single sample (Figure S1; see Additional file 2) to look for correlation between individuals rather than correlation between genes. We can see there is some degree of within experimenter reproducibility, and it seems that when all genes are combined, between experimenter variation is not too different from donor variation. One of the experimental repeats (#4) appears to be an outlier. Therefore, we carried out an analysis both including and excluding this measurement and obtained comparable results.

Based on the MDS analysis of the 13 genes, the two gene clusters showed significant correlation (see Figure 4). We assessed the statistical significance of the two groups by generating randomized datasets that leave the distribution of each gene intact. For each randomization, the gene expression levels of the 145 samples of a gene were randomly permuted. An independent permutation was applied for each gene separately. For the measured MDS plot based on Kendall's correlation (Figure 4), the observed cluster dimensions, defined as the average of all pairwise distances among the genes, were 0.129 and 0.178 for the aforementioned two clusters. For the first cluster (CCL5, IFIT2, IFNA1, IFNA2, IFNB1, IL29), at each randomization, we recomputed the cluster dimension. Based on 4000 randomizations, we found that only one of the cluster dimensions of this six-member cluster was no greater than the observed dimension (0.129), i.e., the six genes were strongly correlated with high level of significance (p-value about 0.0003). We further evaluated the statistical significance of the observed cluster dimension. In each randomization, instead of computing the cluster dimension, we isolated the six-member cluster that gives the smallest cluster dimension. With the same 4000 randomizations, about four of the cluster dimensions of the best six-member clusters were no greater than the observed one, i.e. the group formed a tight cluster with a significant p-value (0.001). When applied to the second cluster (IFIT1, IKBKE, IL6, IRF7, MX1, TBK1), the same analysis showed that the six genes were highly correlated with a significant p-value of 0.0006 and formed a tight cluster with a significant p-value of 0.016.

### Transcription Factor Sharing Analysis

**Figure 5:**
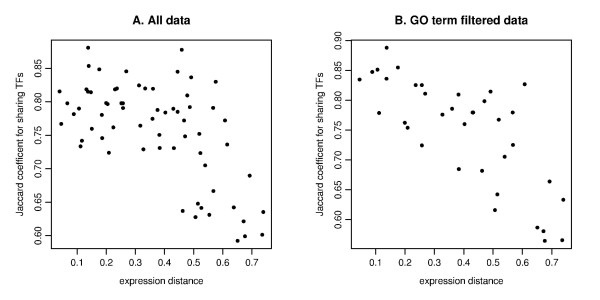
**The scatter plot of the expression distance and TF sharing Jaccard coefficients**. The MDS derived distance for gene expression (on the x-axis) and Jaccard coefficient for sharing TFs (on the y-axis) are plotted for all pairs among 12 genes for **A **(IL29 is not selected due to TF entry) and 9 genes for **B **(IFIT1, IFIT2, IL29 and TBK1 are removed due to no TF entry). The MDS plot is based on the Kendall's correlation coefficient (See Figure 4E). **A**. Pearson's correlation is *r *= -0.606 for all data. **B**. The computation is based on GO filtering for the three immune response related categories (GO:0009615, GO:0002376, GO:0005132). Pearson's correlation is *r *= -0.753.

We investigated whether the sharing of transcription factors (TFs) could be responsible for the clustering. Current studies showed many immunological response genes in mice could be categorized based on their expression dependence on transcription factor IRF-3 [18]. However the underlying gene expression regulation mechanisms were difficult to infer. In agreement with reference [18], we observed that 5 genes (CCL5, IFIT2, IFNA1, IFNB1, IL29) in the first cluster were IRF-dependent response genes [18]. This observation motivated our hypothesis that proximity in dissimilarity space results from shared regulatory components such as TFs. TFs for each of the 13 genes were generated using TF site motif analysis constrained by human-chimp phylogenetic conservation (see Methods). The TF analysis was only based on 12 genes as IL29 has no-entry, probably because of false negatives in the TF prediction algorithm. We used Jaccard coefficients to assess the extent of sharing of TFs between pairs of genes (see Methods). A negative Pearson's coefficient of *r *= -0.606 was found for all pairs of genes for the correlation between gene expression distance (MDS distance) and Jaccard coefficient (Figure 5A), while the value was *r *= -0.422 if we used the actual one minus Kendall's correlation for gene distance. These results were not changed when the analysis was extended to include more deeply rooted phylogenetic conservation. However, due to the high false positive rate in gene to TF mapping, we introduced additional constraints and used a reduced gene to TF mapping table where the gene and the TF must belong to the three immune response related gene ontology (GO) categories (GO:0009615-Immune system process, GO:0002376-Response to virus, GO:0005132-IFNA and IFNB receptor binding). After this GO filtering, the reduced table contained only 9 genes since IFIT1, IFIT2, IL29 and TBK1 have no TF entry. Pearson's coefficient for the MDS distance and Jaccard coefficient was now *r *= -0.753 (Figure 5B), in comparison with *r *= -0.591 for Kendall's correlation based distances. Thus, the negative correlation between MDS distance and Jaccard coefficient was significantly strengthened when the GO constraints were taken into account, presumably through their reduction of the false positive rate of the TF prediction algorithm. The negative correlation confirmed that the closer in distance two genes are in the MDS plot, the more TFs they shared.

### Transcription Factor Enrichment Analysis

Since proximity in dissimilarity space was shown to positively correlate with the fact that gene pairs share TFs, we asked whether genes belonging to one group have in common a number of TFs to a larger degree than the genes that do not belong to it. As a methodology we used TF enrichment analysis. The approach is shown schematically in Figure 1C. We considered the following formulation of the enrichment procedure. For a transcription factor, let *k*_1 _(or *k*_2_) be the number of genes in the cluster (or in the remaining genes) that are bound by this TF. An enrichment score *s *can be defined as the binding fraction difference, *s = k*_1_/*n*_1_-*k*_2_/*n*_2_, where *n*_1 _(or *n*_2_) is the number of genes in the cluster (or outside of the cluster). For a fixed *k *= *k*_1 _+ *k*_2_, the score *s *is a strictly increasing function of *k*_1_, and therefore the p-value for the enrichment score of *s *can be computed by the hypergeometric distribution for *k*_1_. The right sided p-value for score *s *is:

where *n *= *n*_1 _+ *n*_2_, *k *= *k*_1 _+ *k*_2_, and *S *and *K*_1 _denote the random variables associated with observed *s *and *k*_1_, respectively. The p-value is not corrected for multiple comparisons as our goal is not to make individual statements whether some cluster is enriched for some specific transcription factor, but to identify which transcription factors can enter the computation of the total significance score (TSSc). For each cluster, TSSc is summarized by adding up all significant enrichment scores,

where *s*_*i *_and *p*_*i *_are the score and p-value computed for transcription factor *i*. In almost all cases, the condition *p*_*i *_≤ 0.05 ensures a nonnegative score *s*_*i *_so TSSc takes into account all significant scores. A cluster's TSSc measures the extent to which genes in the cluster were co-regulated by transcription factors. For example, when evaluating a cluster of size 2 taken from the 12 genes (IL29 was removed because it lacks a TF entry), for which *n*_1 _= 2 and *n*_2 _= 10, a p-value no greater than 0.05 occurs only when *k*_1 _= 2 and *k*_2 _= 0, i.e. the TF should bind to all genes in the two-member cluster, but not to any gene outside of it. The results of the enrichment analysis are listed in Table S4A [see Additional file 2]. For GO term filtered data (see the TF sharing analysis section), the TF enrichment analysis is only based on 9 genes since IFIT1, IFIT2, IL29 and TBK1 have no TF entry, and the results are listed in Table S4B [see Additional file 2]. For the two clusters identified in the MDS plot, we are left with two four-member clusters (CCL5, IFNA1, IFNA2, IFNB1) and (IKBKE, IL6, IRF7, MX1) after we removed the aforementioned no TF entry genes. In Table S5 [see Additional file 2], among all 126 possible four-member clusters, the greatest TSSc of 4.8 belongs to (CCL5, IFNA1, IFNA2, IFNB1). The next greatest TSSc belongs to (IKBKE, IL6, IRF7, MX1). Thus the two clusters identified by MDS are most likely to be coregulated in terms of TSSc with a p-value of 1/126 = 0.008, and 2/126 = 0.016, respectively, which is consistent with the randomization results described earlier.

For the GO term filtered TF enrichment analysis, we checked the relation between expression distance as given by MDS and the total significance score TSSc. The correlation for all pairs is relatively weak (*r *= -0.329), which is probably due to many zero entries (Table S4B), in comparison with the correlation between MDS and TF sharing (*r *= -0.753). The correlation for the four-member clusters between the cluster dimension (defined as the average distance among all pairs of genes) and the TSSc is somewhat stronger (*r *= -0.456). This relatively weak correlation with MDS distance for the TF enrichment analysis is probably due to the fact that the genes in our analysis are biologically related, which tends to keep at low values the enrichment scores calculated for genes belonging to a cluster.

The TF enrichment analysis results, when compared with those of MDS clustering, need to be interpreted with caution, even when one ignores the presumed occurrence of many false positives. The analysis described above leads to two groups of 4 TFs each in which each of the two clusters is enriched relative to the other. These TFs however, despite the GO filtering by the main category of Immune system process (the others are Response to virus, and IFNA and IFNB receptor binding), do not appear to belong directly to the NDV immune response. Their appearance reflects the fact that across a wide spectrum of immune responses that includes cancer, the genes in the two clusters considered are effectively enriched, and in such a way that their total enrichment scores are larger than those associated with any cluster of four genes chosen from the set of 9 genes (see Table S5). To search for TF enrichment with a set of TFs with a presumed connection to the NDV immune response, we considered a list based on the transcriptional program in macrophages [19], and pathogenic [20] and common responses [21] (See Methods for details). This list includes 67 TFs and 98 motifs. An enrichment analysis gives IRF1 for cluster (CCL5, IFNA1, IFNA2, IFNB1), and CREM, E2F1, E2F6, E2F7, STAT1, STAT2 for cluster (IKBKE, IL6, IRF7, MX1). The results here are more attuned to the experimental situation at hand. In particular STAT1,2 are directly involved through ISGF3 in the transcription of IRF7 and MX1. For other expected TFs in the NDV immune response such as IRF3 or NF-kB (CCL5, IFNB1) that are part of the list, they bind to motifs in the promoter regions of genes in both clusters, have therefore very low enrichment scores, and do not show up in the final result. Thus enrichment analysis has limitations for interpreting the measured cluster structure. One reason is presumably that the relatively small number of genes central to the NDV immune response that are measured at 10 hours after infection are all more or less synchronously up-regulated, which makes it hard to distinguish between clusters unless enrichment encompasses categories of stimulation and cell types that go beyond the confines of the experiment proper. While our focus here was on TFs with known binding site preferences defined in TRANSFAC, future work could apply ab initio methods on these gene clusters to define novel TF candidate binding sites [22].

### Web Tool for Coregulation Analysis

In order to facilitate the use of our methodology by other researchers, we have developed an interactive web-based correlation mapping tool called CorEx. It allows one to combine correlation maps in experimental data, based on hierarchical clustering and MDS analysis, with TF enrichment analysis. A user can choose to constrain the TF prediction by multiple phylogenetic conservations such as chimp, mouse or vertebrates. CorEx uploads the experimental data through the web interface, analyzes correlations and visualizes the hierarchical clustering and MDS. It also presorts genes according to the extent of the corresponding gene correlation. A user can then choose the interesting clusters for TF enrichment analysis. The application will find TFs having binding sites significantly enriched in the promoters of genes forming the submitted clusters. CorEx can be accessed at http://clip.med.yale.edu/corex/corex.php. In addition to the website, we implemented a web service that provides programmatic access to MDS, hierarchical clustering and TF enrichment analysis. This makes it easier to integrate these analyses in existing applications and provides a framework for coregulation-centered analysis of experimental data.

## Conclusions

Our analysis showed that genes which MDS collects into one cluster are coregulated in the sense of sharing transcription factor binding sites, and are as well enriched in TF binding sites when compared with genes outside of the cluster. Our sample of measured genes is small at the start of our investigation, and further reduced by the consideration of experimental uncertainties, followed by GO categories filtering. These steps, designed to obtain statistically meaningful results, are unavoidable precautions that need to be taken all along the investigation. Even the list of TFs needed to study enrichment must be given careful consideration, as we discussed in the corresponding section when replacing the set of TFs in TRANSFAC by an immune response related set. This leads however to a new problem, the fact that some important immune response genes such as NF-kB that bind to many promoter regions, do not show up in the enrichment analysis because of their ubiquity. Our methodology thus highlights a number of issues that beset clustering analysis coupled with TF enrichment, whatever the number of genes investigated. Nevertheless, the overall computational approach developed herein, which is applicable to any type of multivariate data measured in individuals, and is accessible on our web server, provides an efficient hypothesis generator for the mechanisms underlying response variation in humans.

## Methods

### Differentiation of DCs

All human research protocols for this work have been exempted by the IRB of the Mount Sinai School of Medicine due to use of discarded samples not traceable to source. Monocyte-derived conventional DCs were obtained from buffy coats of human blood donors following a standard protocol [23]. Briefly, human monocytes from buffy coats were isolated by Ficoll (Histopaque, Sigma Aldrich) density gradient centrifugation and CD14+ monocytes were immunomagnetically purified by using a MACS CD14 isolation kit (Miltenyi Biotech.). CD14+ monocytes (0.7 × 10^6 ^cells/ml) were later differentiated into immature cDCs after 5-6 day incubation in DC growth media [RPMI Medium 1640 (Gibco), 10% fetal calf serum (Hyclone), 2 mM of L-glutamine, 100 units/ml penicillin, 100 μg/ml streptomycin (Pen/Strep) (Invitrogen), 500 units/ml hGM-CSF (Preprotech) and 1000 units/ml hIL-4 (Preprotech)] at 37°C.

### Virus preparation and viral infection

The recombinant Hitchner strain of Newcastle Disease Virus (rNDV/B1) was prepared and aliquots of allantoic fluid were harvested as previously described [24]. NDV virus stock was titered by infection of Vero cell plates and identification of viral growth by the addition of monoclonal antibodies specific for NDV HN protein (Mount Sinai Hybridoma Core Facility) followed by addition of anti-mouse IgG-FITC and visualization using fluorescent microscopy. Titered NDV stock was diluted in Dulbecco's Modified Eagle Medium (DMEM) and added directly into pelleted DCs at a multiplicity of infection (MOI) of 0.5 prepared as previously described [25]. After incubation for 30 minutes at 37°C, fresh DC growth medium was added back to the infected DCs (1.0 × 10^6 ^cells/ml). Virus free allantoic fluid was added to additional tubes of cells to serve as a negative control.

### qPCR

Ten hours after infection by NDV, DCs were pelleted and total RNA was extracted. The mRNAs of 34 genes and beta-actin were assayed by two step real time RT-PCR. The dried sample total RNA was resuspended into 12 μl DEPC water with 40 ng/μl dT (18 bp) primer. After heating for 10 min at 65°C and placing on ice for 2 min, each sample was mixed with 8 μl RT reaction mix (1 μl Stratscript (Stratagene, Texas) reverse transcriptase (50 U/μl), 2 μl 10× Stratscript buffer, 1 μl 10 mM dNTPs, 4 μl DEPC water) and heated for 2 hrs at 42°C then heated for 15 min at 70°C. The expression levels of 34 genes were determined by real-time PCR using the ABI Prism 9700 HT (Applied Biosystems). All real-time PCR assays were carried out in triplicate in a reaction containing: 1× Jumpstart Taq (Sigma-Aldrich Co.) buffer; 5 mM MgCl_2_; 0.2 mM each dNTPs (dUTP replacing dTTP); 0.5 μM each primers; 0.5 × SYBR Green (Invitrogen); 0.0125 U/μl Jumpstart Taq polymerase; cDNA template; final volume 10 μl. Cycling conditions for all genes were: 95.0°C for 2 min, followed by 40 cycles of 95.0°C for 15 sec, 55.0°C for 15 sec and 72.0°C for 30 sec. The PCR primer sequences are listed in Table S6 [see Additional file 2].

### Hierarchical clustering and MDS

The mRNA copy numbers were collected from 145 samples described above, and were then transformed by the log_2 _function and further normalized by the subtracting the median. The matrix for each of the three types of correlations was computed using the statistical programming language R [26,27] by the function ***cor***. The detailed mathematical formulation is described in [16] or in the Supporting Information, Correlations Computations [see Additional file 2]. One defines the dissimilarity by one minus the correlation; the dissimilarity matrix can be used to construct the dendrogram for the hierarchical clustering by the R function ***hclust ***and the two dimensional coordinates of the genes for the multidimensional scaling (MDS) by the R function ***cmdscale***. All R functions are run with version 2.8.0.

### TF mapping

For predicting TFs binding to regulatory regions of the studied genes, we considered three gradually decreasing levels of phylogenetic conservation: (i) across rat, mouse, dog and human, (ii) across mouse and human, (iii) across chimp and human. Details of chimp and human conservation are as follows, with the others being similar: We used the aligned regions between human (version hg18) and chimp (version panTro2), which can also be downloaded from the UCSC genome bioinformatics website [28]. The promoter region [-2k, 0] from the transcription starting site (TSS) is obtained for each human gene, where TSS is defined by the March 2006 refGene table [29]. The TRANSFAC MATCH [30] with a cutoff chosen to minimize the sum of false positives and negatives is used to identify putative transcription factor binding sites for the human promoter region and the aligned chimp promoter region for all vertebrate transcription factor matrices in the 2009.1 release of TRANSFAC [31]. The putative binding sites were considered to be phylogenetically conserved if matches were found in both human promoter regions and aligned chimp promoter regions of the same gene and no gaps were present in the human chimp alignment. Only three of the genes in this study (MX1, IRF7 and CREM) were associated with multiple TSSs. In these cases, we used the union of putative transcription factor binding sites from all of the alternate promoters. Vertebrate TRANSFAC matrices were included only if they could be linked to a HGNC [32] gene symbol, either directly or by an alias. A TF gene is listed as a presumed connection to the NDV immune response only if its gene symbol or its alias also appeared in Table S5 of reference [10], Table S8 of reference [11] or Table 1 of reference [12].

### Jaccard coefficient for genes sharing TFs

Let *S*_1 _and *S*_2 _be the sets of transcription factors that bind to genes g1 and g2, respectively. The Jaccard coefficient for the two genes sharing TFs is , the ratio of the numbers of TFs in the intersection set of *S*_1 _and *S*_2 _to the number of TFs in the union set of *S*_1 _and *S*_2_.

### Web tool

The ***CorEx ***application consists of two interrelated elements: a CorEx analyzer and a front end web-interface. CorEx is scripted in PERL, calls R subroutines for the analysis, and presents the results via the Common Gateway Interface. The CorEx analyzer retrieves input data through the web-interface to generate MDS plots and a corresponding correlation matrix. The hierarchical clustering plot is generated as well if requested (see corresponding section of Methods). The analyzed result is displayed through the web-interface. The CorEx source code is available through the GNU General Public License and accessible at SourceForge upon request. The home page of CorEx is http://clip.med.yale.edu/corex/corex.php.

The web service provides an interface to MDS, hierarchical clustering and TF enrichment analysis that can be accessed through the JavaScript Object Notation (JSON). This widely supported standard is based on a subset of the JavaScript Programming Language, Standard ECMA-262 3rd Edition - December 1999. Image versions of a requested analysis can be retrieved in several graphics formats, including rasterized formats (e.g., jpg) and vector graphics formats (e.g., SVG and PDF) accompanied by plain text files listing analysis's details. Additional details are provided in FAQs on the web site.

## Competing interests

The authors declare that they have no competing interests.

## Authors' contributions

SS, JW, YG and GN designed the overall computational approach. JH and JW conceived the co-expression experiments which were performed by JH andMK. SS and JW planned the array experiments which were performed by JS. YG proposed and implemented analysis with contribution from GN. GN developed the Web tool. FH refined the analysis and oversaw the whole project. GN, YG and SS wrote the manuscript with contributions from all authors. JD and SK generated the phylogenetically-constrained TF binding site data. All authors read and approved the final manuscript.

## Supplementary Material

Additional file 1 **Supporting Information: Table S1.** The copy numbers of mRNA for all 34 genes. Click here for file

Additional file 2 **Supporting Information: Correlations computations.** Supporting tables: Table S2. The summary table of the gene expression data for all 34 genes. Table S3. Three pairwise correlation among the 13 filtered genes. Table S4. TF enrichment analysis for pairs of genes. Table S5: TF enrichment analysis for Go filtered four-member clusters. Table S6: The PCR Primer for all 34 genes and beta-actin. Supporting figures Figure S1: The MDS plot for the 145 donors and the six experimental repeats. Click here for file
