# Supplementary material for: Coregulation mapping based on individual phenotypic variation in response to virus infection
Source: Immunome Res. 2010 Mar 18;6:2. doi: 10.1186/1745-7580-6-2 (PMC3161383; doi:10.1186/1745-7580-6-2)
Supplement: Additional file 2 — Supporting Information: Correlations computations. Supporting tables: Table S2. The summary table of the gene expression data for all 34 genes. Table S3. Three pairwise correlation among the 13 filtered genes. Table S4. TF enrichment analysis for pairs of genes. Table S5: TF enrichment analysis for Go filtered four-member clusters. Table S6: The PCR Primer for all 34 genes and beta-actin. Supporting figures Figure S1: The MDS plot for the 145 donors and the six experimental repeats. [file 1745-7580-6-2-S2.DOC]

**Coregulation Mapping Based on Individual Phenotypic Variation in Response to Virus Infection**

**Supporting Information**

**Correlations computations**

 For pairs of quantities (*x_i_*, *y_i_*), *i*=1,..,*n,* the *n* is the number of samples, and is equal to 145 for our data in the main text. **Pearson**’s correlation (*r*) is given by

,

where$\bar{x}$and $\bar{y}$ are the sample average of *x*’s and *y*’s respectively.

Let *R_i_* be the rank of *x_i_* among all the *x*’s, *S_i_* be the rank of *y_i_* among all the *y*’s. If some of *x_i_*’s (*y_i_*’s) are identical, all these ties are assigned the mean of the ranks that they have had if their values had slightly been different. **Spearman**’s correlation ($\rho$) captures monotonic correlation, and is defined the same as the Pearson’s correlation except that the (*x_i_*,*y_i_*) is replaced by the ranks (*R_i_*,*S_i_*). Let $\bar{R}$ and $\bar{S}$ be the sample average of *R’s* and *S’s* respectively. The formula for Spearman’s correlation is given by

.

**Kendall**’s correlation ($\tau$) is more non-parametric than Spearman’s. Instead of using the numerical difference of ranks, it uses only the relative ordering of ranks (values). In this case, the data do not have to be ranked at all, as the relative ordering (higher, lower, or the same) of ranks is equivalent to that of the values. Now consider all *n*(*n*-1)/2 pairs of data points. (i) A pair is called *concordant* if the relative ordering of the ranks of two *x*’s (or the two *x*’s themselves) is the same as the relative ordering of the ranks of two *y*’s (or the two *y*’s themselves). (ii) A pair is called *discordant* if the relative ordering of two *x*’s is opposite from that of two *y*’s. (iii) If there is a tie in the ranks of two *x*’s but not in the ranks of two *y*’s, the pair is called “*extra y pair*”. (iv) We can also define “*extra x pair*” be similarly. (v) If the tie is in both the *x*’s and *y*’s, the pair is ignored in the computation. Kendall’s correlation ($\tau$) is defined by

$$\tau=\frac{concordant-discordant}{\sqrt{concordant+discordant+extra\_x}\sqrt{concordant+discordant+extra\_y}}$$

**Supporting tables**

**Table S1. The copy numbers of mRNA for all 34 genes**. The measurements are obtained by qPCR and are plate corrected. The top block is for the expression levels for all 134 samples, the bottom block for 6 repeats for checking experimental variation.

**Table S2. The summary table of the gene expression data for all 34 genes**. The median and MAD are given for the copy numbers, and the mean and standard deviations are given for the log_2_ copy numbers. The first four columns are for the population data of 145 samples. The next two columns are for the 6 repeats for checking experimental variation. The last column indicates the % of donor population variation that is contributed by the experimental variation. The four blue genes with a crossed line have low gene expression, and the entry for the last column is colored red when the experimental variation is significantly smaller than the population variation (p-value of the F-test is no greater than 0.05). The median and MAD (median absolute deviation) indicate the center and spread of the non-Gaussian distribution of the mRNA copy numbers across 145 samples. The median is defined as the point such that 50% of the data fall below or above it. MAD is defined as $1.48\times median(|x_{i}-m|)$, where m=median(x_i_), and x_i_ are the mRNA copy numbers from the 145 samples.

**Table S3**. **Three pairwise correlation among the 13 filtered genes**. Pearson’s (**A**), Spearman’s (**B**) and Kendall’s (**C**) correlation coefficients for all pairs of genes are shown. Since the matrix for each correlation is symmetric, only the lower triangle is displayed. All of p-values for the correlations are less than 5x10^-4^.

**Table S4**. **TF enrichment analysis for pairs of genes**. The TF prediction was phylogenetically constrained by human-chimp conservation. **A)** All data set, **B)** GO term filtered. Only the entry with non-zero TSS’s are listed and the list is ordered according to TSS. The two genes in the pair are linked with a dot in the table.

**Table S5: TF enrichment analysis for Go filtered four-member clusters**.

**Table S6: The PCR Primer for all 34 genes and beta-actin**.

Table S1 – See attached Excel File

Table S2

Table S3

(A): Pearson's correlations

(B): Spearman's correlations

(C) Kendall's correlations

Table S4

1. All data
2. GO term filtered data

Table S5

Table S6

|  |  | **ACCESSION#** | **SEQUENCE (5'-3')** |
| --- | --- | --- | --- |
| **1** | B2MicroGlo | NM_001101 | GTGGACTTGGGAGAGGACTG |
|  |  | NM_001101 | ACTGGAACGGTGAAGGTGAC |
| **2** | CASP8 | NM_001228 | TCCAAGCAGAGATGAAAGAG |
|  |  | NM_001228 | ATAAGCTCTCCCCAAACTTG |
| **3** | CCL4 | NM_002984 | ATCCCCATAGGACACTTATC |
|  |  | NM_002984 | CACATCTCCTCCATACTCAG |
| **4** | CCL5 | NM_002985 | AAGCTCCTGTGAGGGGTTGA |
|  |  | NM_002985 | TTGCCAGGGCTCTGTGACCA |
| **5** | CCR7 | NM_001838 | AGGTTTTCAGTCCCTGTGAC |
|  |  | NM_001838 | TGACATGCACTCAGCTCTTG |
| **6** | CD86 | NM_175862 | TGTTAGAAACTAGCCAGGTG |
|  |  | NM_175862 | GTCTCTGCCCAAACATAAAG |
| **7** | DICER1 | NM_177438 | AAGGTGCTGTGTTTTGCTTC |
|  |  | NM_177438 | TACTAAAGTCCTCCTGCCAG |
| **8** | IFIT1 | NM_001548 | GACCTTGTCTCACAGAGTTC |
|  |  | NM_001548 | TCGGAGAAAGGCATTAGATC |
| **9** | IKBKE | NM_014002 | GAGTGAGGGAGAGCCAAAGG |
|  |  | NM_014002 | CCAGGGCAGTAGGTCAAAC |
| **10** | IL12A | NM_000882 | CTCCCTAGTTCTTAATCCAC |
|  |  | NM_000882 | GCCACAAAAATCCTCCCTTG |
| **11** | IL28A | NM_172138 | TGGGCTGAGGCTGGATACAG |
|  |  | NM_172138 | TCTGGAGGCCACCGCTGACA |
| **12** | IL28B | NM_172139 | CGTGGGCTGAGGCTGGATAC |
|  |  | NM_172139 | TGGCCCTGACGCTGAAGGTT |
| **13** | IL29 | NM_172140 | GGAGTAGGGCTCAGCGCATA |
|  |  | NM_172140 | GCCTCCTCACGCGAGACCTC |
| **14** | IL6 | NM_000600 | CTGAGGTGCCCATGCTACAT |
|  |  | NM_000600 | AATGCCAGCCTGCTGACGAA |
| **15** | IL8 | NM_000584 | CAACATCACTGTGAGGTAAG |
|  |  | NM_000584 | GTTAAATCTGGCAACCCTAG |
| **16** | IFNA1 | NM_000605 | ATTTCTGCTCTGACAACCTC |
|  |  | NM_024013 | CTGAATGACTTGGAAGCCTG |
| **17** | IFNA2 | NM_000605 | ATTTCTGCTCTGACAACCTC |
|  |  | NM_000605 | TGACAGAGACTCCCCTGATG |
| **18** | IFNAR1 | NM_000629 | CTTGCCCGTATTTTTAGGAC |
|  |  | NM_000629 | GTGAAGAACTACAGCAGGAC |
| **19** | IFNB1 | NM_002176 | ACAGCATCTGCTGGTTGAAG |
|  |  | NM_002176 | GTCAGAGTGGAAATCCTAAG |

|  |  | **ACCESSION#** | **SEQUENCE (5'-3')** |
| --- | --- | --- | --- |
| **20** | IFNG | NM_000619 | GCTATGTTTTCATCAGGGTC |
|  |  | NM_000619 | AGGCAAGGCTATGTGATTAC |
| **21** | CXCL10 | NM_001565 | TGAAGCAGGGTCAGAACATC |
|  |  | NM_001565 | TCCCATCACTTCCCTACATG |
| **22** | IRF7 | NM_001572 | GCTCCAGCTCCATAAGGAAG |
|  |  | NM_001572 | GGTGTGTCTTCCCTGGATAG |
| **23** | IRF9 | NM_006084 | ATTAGCCTTGAGTTCTCCAC |
|  |  | NM_006084 | ATTCTGTCCCTGGTGTAGAG |
| **24** | IFIT2 | NM_001547.3 | TCGTTCCAAGCATACCGTGA |
|  |  | NM_001547.3 | CGTGGGAACCTGGTGACTAA |
| **25** | MX1 | NM_002462 | TGCAAGGTGGAGCGATTCTG |
|  |  | NM_002462 | CGTGGTGATTTAGCAGGAAG |
| **26** | EIF2AK2 | NM_002759 | CATGTCAGGAAGGTCAAATC |
|  |  | NM_002759 | ACTACGTGTGAGTCCCAAAG |
| **27** | DHX9 | NM_001357 | TCTAAAGCCACCTCGGGAAA |
|  |  | NM_001357 | ATGGCGGTGGATATAGCAGT |
| **28** | DDX58 | AF038963 | GGCTTGGGATGTGGTCTACT |
|  |  | AF038963 | AAAGCCTTGGCATGTTACAC |
| **29** | STAT1 | NM_007315 | CCTTTCAATTTTACCTTCAG |
|  |  | NM_007315 | CTTCTCTGGCGACAGTTTTC |
| **30** | TBK1 | NM _013254 | TGGGATCTGGGCACCTTGTA |
|  |  | NM _013254 | TGGTAGAACGGTGGCTACTG |
| **31** | TLR3 | NM_003265 | ACATTCCTCTTCGCAAACAG |
|  |  | NM_003265 | TGAGGCGGGTGTTTTTGAAC |
| **32** | TNF | NM_000594 | GAGGAAGGCCTAAGGTCCAC |
|  |  | NM_000594 | AGTGAAGTGCTGGCAACCAC |
| **33** | TRAM1 | BC_000687 | GGAACAAGATGTGAACACTG |
|  |  | BC_000687 | GTATCGCTACAGAAAGGCTC |
| **34** | TYK2 | NM_003331 | GGATTTAAGGGCTGGATTAG |
|  |  | NM_003331 | AACCAAGAGGGGGATGTCAG |
| **35** | Beta-Actin |  | GTGGACTTGGGAGAGGACTG |
|  |  |  | ACTGGAACGGTGAAGGTGAC |

**Supporting figures**

**Figure S1: The MDS plot for the 145 donors and the six experimental repeats.** For each gene, the log2 copy numbers of 145 donors is normalized such that its median is zero, and the median normalization is also applied to the six experimental repeats. The 151 combined samples are analyzed by the MDS using Kendall’s correlation. The six experimental repeats are colored in red and labeled from 1 to 6, in which (1,2), (3,4), and (5,6) are done by the one of the three experimenters, respectively. Note the close correspondence in measurements 1,2 and 5,6. The distance between measurements 3 and 4 presumably results from experimental error affecting one of the assays.


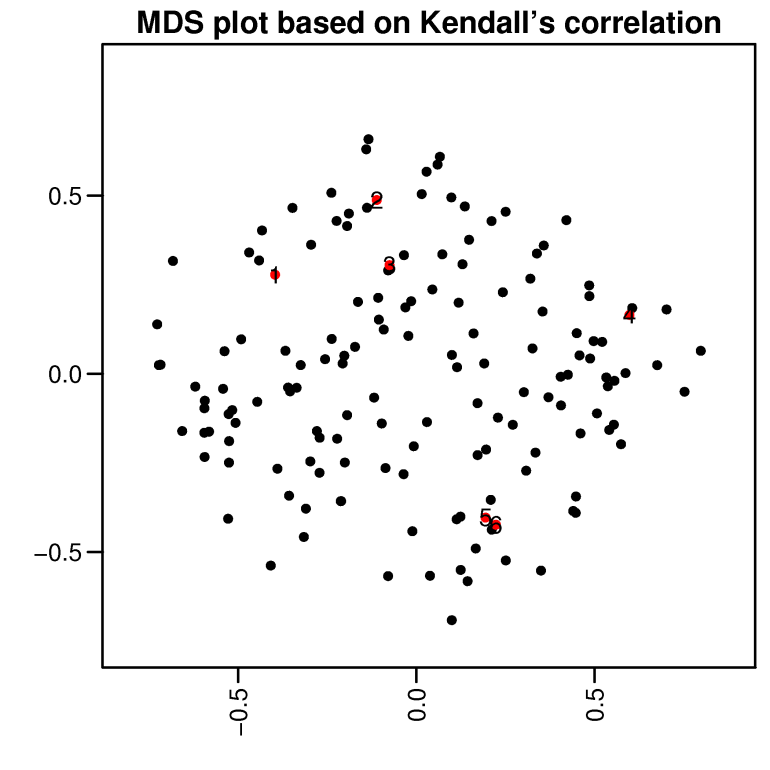
Figure S1
